# Engineering the Chloroplast Genome of Oleaginous Marine Microalga *Nannochloropsis oceanica*

**DOI:** 10.3389/fpls.2018.00439

**Published:** 2018-04-11

**Authors:** Qinhua Gan, Jiaoyun Jiang, Xiao Han, Shifan Wang, Yandu Lu

**Affiliations:** ^1^State Key Laboratory of Marine Resource Utilization in South China Sea, College of Oceanology, Hainan University, Haikou, China; ^2^Key Laboratory of Tropical Biological Resources, Ministry of Education, Hainan University, Haikou, China

**Keywords:** *Nannochloropsis*, plastid transformation, oleaginous microalga, green fluorescent protein, photosynthetic cell factory

## Abstract

Plastid engineering offers an important tool to fill the gap between the technical and the enormous potential of microalgal photosynthetic cell factory. However, to date, few reports on plastid engineering in industrial microalgae have been documented. This is largely due to the small cell sizes and complex cell-wall structures which make these species intractable to current plastid transformation methods (i.e., biolistic transformation and polyethylene glycol-mediated transformation). Here, employing the industrial oleaginous microalga *Nannochloropsis oceanica* as a model, an electroporation-mediated chloroplast transformation approach was established. Fluorescent microscopy and laser confocal scanning microscopy confirmed the expression of the green fluorescence protein, driven by the endogenous plastid promoter and terminator. Zeocin-resistance selection led to an acquisition of homoplasmic strains of which a stable and site-specific recombination within the chloroplast genome was revealed by sequencing and DNA gel blotting. This demonstration of electroporation-mediated chloroplast transformation opens many doors for plastid genome editing in industrial microalgae, particularly species of which the chloroplasts are recalcitrant to chemical and microparticle bombardment transformation.

## Introduction

Microalga-based biochemical factory is regarded as an ideal strategy for sequestering greenhouse gas and producing valuable molecules ranging from therapeutic proteins to biofuels (Tran et al., 2013; Moody et al., 2014). However, few natural strains exhibit the demanding traits as feedstock for biofuel production which have led to a quest for more specific genomic and biological models (Scott et al., 2010; Ge et al., 2014). *Nannochloropsis* spp. have attracted sustained interest from algal biofuels researchers owing to their rapid growth, high amounts of triacylglycerol (TAG) and high-value polyunsaturated fatty acid (FA) and their successful cultivation at large scale using natural sunlight by multiple institutes and companies (Radakovits et al., 2012; Vieler et al., 2012; Wang et al., 2012, 2014; Corteggiani Carpinelli et al., 2014; Lu et al., 2014a,b; Moody et al., 2014; Lu and Xu, 2015; Ajjawi et al., 2017; Wei H. et al., 2017; Zienkiewicz et al., 2017).

Genetic engineering of industrial microalgae provides a viable way to optimize crucial traits for commercial feedstock development (Gimpel et al., 2013; Zhang and Hu, 2014; Wang et al., 2016; Cui et al., 2018). A nuclear transformation method has been developed for *Nannochloropsis* sp. (Kilian et al., 2011; Vieler et al., 2012; Li et al., 2014; Iwai et al., 2015; Kang et al., 2015; Poliner et al., 2017; Xin et al., 2017), which facilitates the manipulation of crucial nodes in oil biosynthesis and the development of the RNA interference (RNAi) (Wei L. et al., 2017) and CRISPR/Cas9 methods (Wang et al., 2016). However, the plastome genetic engineering tools are not yet avaiable for *Nannochloropsis* spp. There are considerable attractions associated with placing transgenes into the plastid genome rather than the nuclear genome (Bock, 2014; Doron et al., 2016), particularly where plastid genomes are engineered to express valuable proteins (e.g., therapeutics proteins) (Tran et al., 2013): (i) high transgene expression levels; (ii) capacity for expressing multigene in artificial operons; (iii) devoid of gene silencing and other epigenetic mechanisms; (iv) higher precise insertion site than nuclear expression (which normally integrate foreign DNA into their nuclear genomes by non-homologous recombination).

Besides the manipulation of plastid genes (with a number of ~100) (Wei et al., 2013), transplastomic technology may utilized to express heterologous genes or gene clusters with economic values (e.g., pharmaceutical proteins) (Mayfield et al., 2007; Rasala et al., 2010). Moreover, ~10% of the nuclear gene products (mainly FA biosynthetic enzymes and photosynthesis related proteins which determine the key features of oleaginous microalgae for biofuel production) are targeted to plastids (Leister, 2003). This further expands the plastome engineering gene repertoire. Thus, transplastomic technology provided fundamental opportunities for rational trait-improvement of microalgae (Bock, 2014).

Although progresses have been made for several reference plants, plastid transformation is still restricted to a relatively small number of species (Bock, 2014). This is mainly due to the fastidious requirements in cell handling to match the methods currently available for plastid transformation (Maliga, 2004). For instance, although microparticle bombardment is a rountine pratice to delivery exogenous DNA into plant or microalgal plastids, it has a rigid requirement to cell diameters of target species (Cui et al., 2014). Genetic manipulation of chloroplasts of small-size microalgal species is intractable due to the limitation of availablity of golden particles (of which the smallest diameter is 0.6 μm). Therefore, biolistic plastid transformation have only been developed for a few micoalgal species (exclusively for species with relatively large cell sizes and huge chloroplasts), e.g., *Chlamydomonas reinhardtii* (with a diameter of ~10 μm) (Boynton et al., 1988), red alga *Porphyridium* sp. (with a diameter of ~15 μm) (Lapidot et al., 2002) and green alga *Platymonas subcordiformis* (with a diameter of ~15 μm) (Cui et al., 2014). However, as for most industrial microalgal species of which the diameters are approximately a few microns (e.g., *Nannochloropsis* sp. and *Chlorella* sp., both with a diameter of ~2 μm), plastome genetic engineering tools have not yet been developed. Polyethylene glycol (PEG) treatment of protoplasts provides an alternative way for chloroplast transformation (Golds et al., 1993). However, as all protoplast-based methods, PEG-mediated protoplast transformation requires removal of the cell wall prior to transformation (or, alternatively, use of cell wall-deficient mutant strains), which makes the procedures technically demanding, labor intensive, and time consuming (Bock, 2015). Even worse, protoplast preparation is always intractable to most microalgal species of which the cell wall is complex (Maliga and Bock, 2011). Therefore, research and development of plastid biotechnology remain challenge for most industrial microalgae.

During the creation of nuclear mutagenesis library for industrial oleaginous microalga *Nannochloropsis oceanica*, we found that antibiotic constructs were inserted into the plastid genome by electroporation. A similar phenomenon has also been documented in *C. reinhardtii* (Zhang et al., 2014; Li et al., 2016). Therefore, to probe the potential of applying electroporation in chloroplast transformation, employing *N. oceanica* as a model, a simple and rapid approach for chloroplast transformation was developed for *N. oceanica*.

## Materials and methods

### Gene cloning and vector construction

Genomic DNA was extracted using Plant Genomic DNA Extraction Kit (Omega, China) following a described procedure (Lu et al., 2009, 2010). *N. oceanica* endogenous *chlL* gene fragments, *rbcL* promoter and *psbA* terminator were amplified using sequence specific primers (Supplementary Table 1). The upstream and downstream fragments of *chlL* gene were subcloned into pBluescript SK vector (Stratagene, USA) using *Kpn*I, *Xho*I and *Sac*I, *Bam*HI sites, respectively. The *rbcL* promoter and *psbA* terminator were subsequently ligated into the resulting vector between the *Xho*I, *Hind*III and *Eco*RV, *Eco*RI sites, respectively. The codon optimized *gfp* gene was synthesized by Sangon Biotech (Shanghai, China) and was ligated into the above vector with a *Hind*III restriction site at the 5′ end and an *Eco*RV site at the 3′ end of the coding region. All enzymes were commercially available from New England BioLabs (NEB, UK). The obtained vector was nominated as pMEMc1. The zeosin resistance (BLE) gene was amplified from vector pSP124 using primers BLE-F and BLE-R (Supplementary Table 1). The *gfp* gene of pMEMc1 was substituted by the *BLE* gene and generated vector pMEMc2.

### Strains, transformation, and growth conditions

*N. oceanica* was inoculated into modified f/2 liquid medium, which was prepared as early description (Gan et al., 2017). The cells were grown in liquid cultures under continuous light (~50 μmol photons m^−2^ s^−1^) at 25°C. Transformation was conducted as description with minor modification (Wang et al., 2016; Xin et al., 2017). Vectors were linearized by restriction digestion, and purified and concentrated by ethanol precipitation. Microalgal cells at early log phase were harvested by centrifugation at 5,000 g for 5 min at 4°C. Cells were washed with sorbitol at 4°C. For each transformation reaction, 4 × 10^8^ cells were mixed with 1 μg transforming cassette DNA. The mixture was added into a cuvette (Bio-Rad, 2 mm) and pulsed using GenePulse Xcell^TM^ (BioRad) apparatus with 12 kV cm^−1^ field strength, 50 μF capacitance, and 600 Ohm shunt resistance. The cells were immediately transferred into fresh f/2 medium and recovered under dim light for 48 h. For pMEMc1 transformants, GFP fluorescence was observed at indicated intervals while for pMEMc2 transformants, cells were plated on solid f/2 with 2.5 μg ml^−1^ zeosin (Solarbio, China) and colonies appeared after ~4 weeks.

### GFP expression detection of pMEMc1 transformants

An Olympus BX51 microscope (Olympus, Japan), fitted with epifluorescence and differential interference contrast (DIC) optics, was used to visualize pMEMc1 transformants grown in liquid. Images were generated by either DIC or epifluorescence (excitation, 488 nm; emission, 520 nm) optics. An Olympus FluoView^TM^ FV1000 system was used to obtain laser confocal scanning microscope (LSCM) images of pMEMc1 transformants. Excitation at 488 and 559 nm were used for GFP and chlorophyll autofluorescence, respectively.

### Genotyping of pMEMc2 transformants

**Figure 1:**
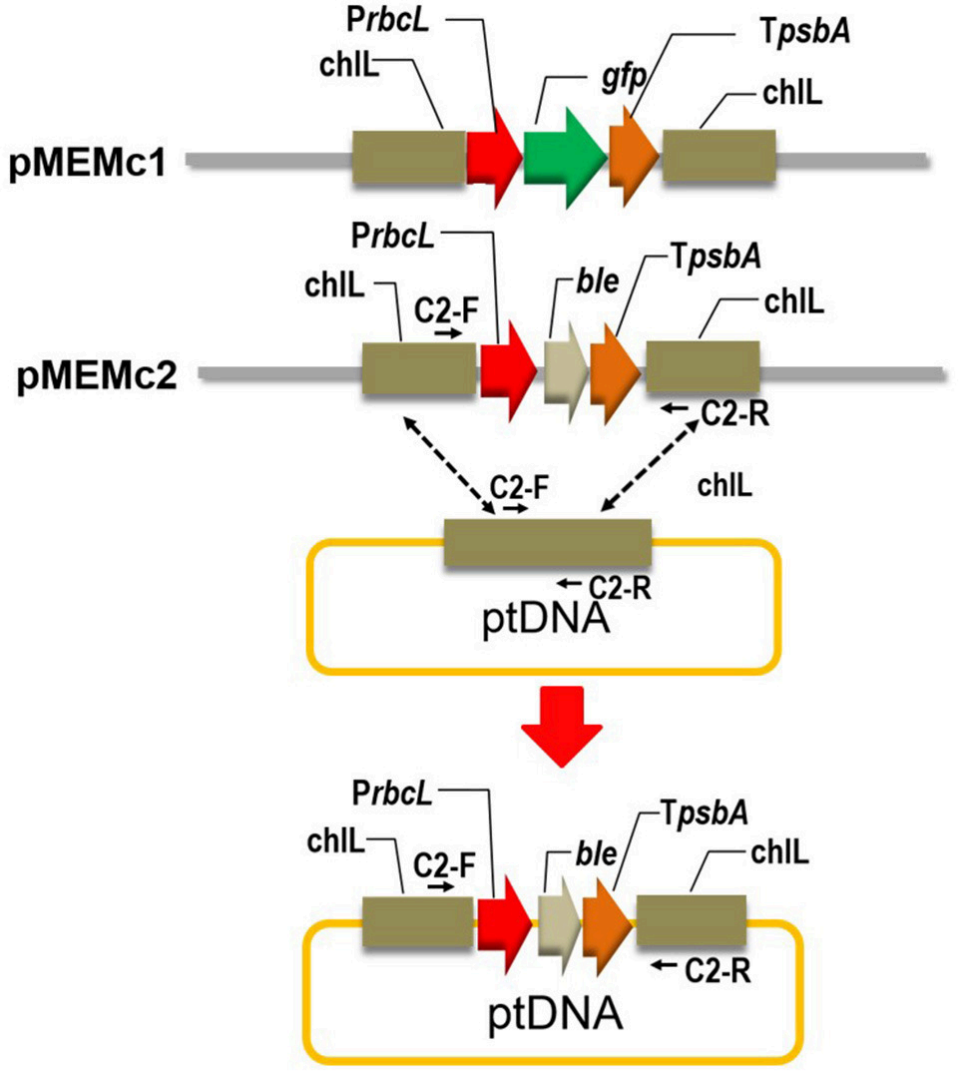
Construct map of homologous recombinant vector in *N. oceanica* chloroplast. P*rbcL*, promoter of large subunit of RuBisCO gene (*rbcL*); T*psbA*, 3′ flanking sequence of gene encoding the D1 protein of Photosystem II (*psbA*); *gfp*, green florescence protein gene; chlL, light-independent protochlorophyllide reductase subunit; *ble*, zeosin resistance gene. Primers designed to examine the homoplasmic integration of the transforming cassette is indicated (c2-F and c2-R; Supplementary Table 1).

Approximately 10 mL cultures of wild-type and pMEMc2-transformed cells were harvested by centrifugation (7,000 rpm, 3 min at 4°C). The cell pellet was washed twice, and then genomic DNA was extracted. Genomic PCR was used to confirm the homoplasmic integration of the vector into the plastid genome of transgenic lines. With the primers crossing *chlL* gene regions (c2-F and c2-R; Supplementary Table 1 and Figure 1), 0.6 and 1.9 kb PCR products were expected to be detected for wild type and homoplasmic transformed cells (all copies of the chloroplast genome contained the *ble* gene), respectively. By contrast, for cells harboring heterogeneous chloroplast genomes, PCR amplification should generate both of the two bands (i.e., 0.6 and 1.9 kb DNA bands). All PCR fragments were purified (Cycle-Pure Kit, Omega, China) and sequenced (Sangon, China). Integration events were further analyzed by DNA gel blotting using non-radioactive DIG-containing ble gene probes (PCR DIG probe synthesis kit; Roche Diagnostics). The *ble* gene probes were labeled with DIG-dUTP using a pair of primer (BLE-F and BLE-R; Supplementary Table 1). Genomic DNA was digested with restriction enzyme *Hind*III or *Pst*I, separated on 0.8% agarose gels, blotted, hybridized, and visualized.

## Results

### Design and construction of destination vectors

To facilitate visualization of protein expression, the green florescence protein gene (*gfp*) was used as a reporter (Figure 1) and codon-optimized based on the featured codon bias and a high AT content (66.4%) of the *N. oceanica* chloroplast genome (Wei et al., 2013). The GFP gene was driven by an endogenous promoter (large subunit of RuBisCO, *rbcL*) and terminated by an endogenous terminator (3′ flanking sequence of gene encoding the D1 protein of Photosystem II, *psbA*) (Figure 1 and Supplementary Dataset 1). This expression cassette contains homology to the chlorophyll synthetic gene light-independent protochlorophyllide reductase subunit (*chlL*) region of the *N. oceanica* chloroplast genome. Transforming cassette will insert into the *chl*L locus by homologous recombination in expected transformants (Figure 1). Transformation construct harboring *gfp* gene (*chlL*-*rbcL*-*gfp*-*psbA*-*chlL*) were cloned into the plasmid pBluescript SK(-) and nominated as pMEMc1. The coding sequence of GFP in the vector was substituted with that of BLE gene and the resulting vector was designated as pMEMc2 (*chlL*-*rbcL*-*ble*-*psbA*-*chlL*; Figure 1 and Supplementary Dataset 2).

### Introduction of reporter genes into the chloroplast genome

**Figure 2:**
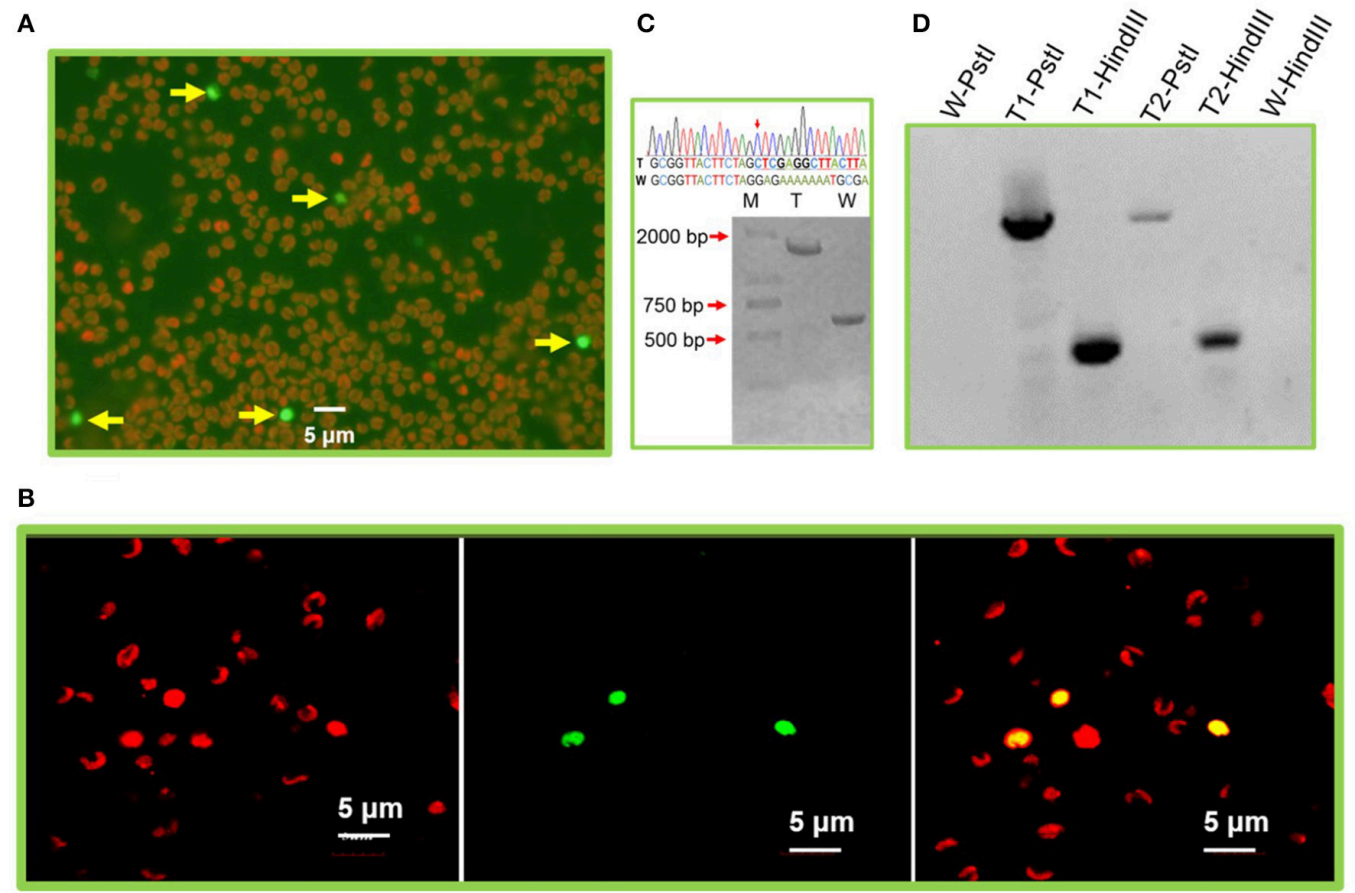
Exogenous gene expression in the *N. oceanica* chloroplast. **(A)** Microscopy images of GFP signals from representative microalgal cells transformed by pMEMc1. The fluorescent micrographs show the GFP expressing cells with green color (excitation: 488 nm, emission: 500–545 nm) and wild type cells with auto red fluorescence of chlorophyll (excitation: 559 nm, emission: 570–650 nm). **(B)** Laser confocal microscopic observation of *N. oceanica* pMEMc1 transformants. Left, chlorophyll fluorescence; middle, GFP fluorescence; right, merged image. **(C)** PCR amplification of wild-type cells and pMEMc2 transformants genomic DNA using c2-F and c2-R primers. PCR product of wild type cells generates a single 0.6 kb DNA band. Homoplasmic cells harbors a 1.3 kb transforming constructs (*rbcL*-*ble*-*psbA*) and was expected to generate a single 1.9 kb DNA band. **(D)** DNA gel blot of pMEMc2 transformants. Wild-type cells was used as a control. Genomic DNA was digested with restriction enzyme *Hind*III or *Pst*I and blotted with DIG-dUTP labeled *ble* gene probes. W, Wild-type cells; T, pMEMc2 transformants; -PstI, genomic DNA digested *Pst*I; -HindIII, genomic DNA digested *Hind*III.

To probe the proper *in vivo* functioning of selected plastid promoters and terminators in *N. oceanica*, we started by transforming pMEMc1 cassette into *N. oceanica* wild-type strain by electroporation. Fluorescence microscopy of representative cells revealed that GFP protein was delivered into and expressed in *N. oceanica* (excitation: 488 nm, emission: 500–545 nm; Figure 2A and Supplementary Video 1). The number of transformants expressing GFP increased, and reached the highest levels 3 days after pulse. Laser confocal microscopy further confirmed the *in vivo* GFP expression in *N. oceanica* (Figure 2B). Despite of a low possibility of functioning in nuclear expression of these utilized plastid promoter and terminator, we cannot exclude the possibility that GFP expressed in nuclear instead of chloroplast.

### Site-specific integration of transgenes into the chloroplast genomes

To validate the homologous recombination of the exogenous constructs into chloroplast genomes, pMEMc2 was transformed into the wild-type *N. oceanica*. Appropriately 4 × 10^8^ *N. oceanica* cells were used for each pulse with 1 μg transforming cassette DNA. The cells were recovered under dim light for 48 h before being plated on f/2 plates containing zeocin (2.5 μg/ml). Approximately eight transformed colonies appeared on the selective plates which translated to a transformation frequency of about 2 × 10^−8^ μg^−1^ DNA. Two transformants were selected and analyzed for integration and homoplasmicity after multiple rounds of streaking of single colonies under zeocin-resistance selection (no less than four rounds each of which took approximately a month). Genomic PCR and sequencing confirmed that homoplasmic strains (all copies of the chloroplast genome contained the *ble* gene) were obtained and the *rbcL*-*ble*-*psbA* constructs (~1.3 kb) were integrated into all chloroplast genomes through homologous recombination of *chlL* regions (Figure 2C and Supplementary Dataset 3). Control reactions using genomic DNA from wild type as templates yield PCR products with a length of ~0.6 kb because they did not contain the vector sequences (Figure 2C and Supplementary Dataset 4). It is not clear whether a single insertion occurred in transformed cells (or whether random insertions happened in transformant genome). Further analysis of integration events was performed by DNA gel blot where a single band was observed by using *ble* probe in either *Hind*III or *Pst*I digested genomic DNA of transformants (Figure 2D). Altogether, a stable and targeted transgene integration within the plastid genome was mediated by electroporation.

## Discussion and conclusion

Chloroplast transformation was generally achieved by the biolistic process and ocassionally by PEG-mediated method (Maliga, 2004). However, neither of them is competent for most industrial microalgae. The major bottleneck is the inaccessibility of competent methods for DNA delivery into various microalgae with myriad cell size, complex, and largely unknown cell wall components. Thus, microalgae amenable to plastid transformation have been confined to limited species (Doron et al., 2016). Electroporation, which is normally used for nuclear transformation, was found to be capable of delivering exogenous DNA into plastid genome of *N. oceanica* (Supplementary Dataset 5) and *C. reinhardtii* (Zhang et al., 2014; Li et al., 2016).

Therefore, the aim of this study is to demonstrate the capacity of electroporation-mediated method in the development of transplastomic technology for species with small cell size and unknown cell-wall components. Using a plastid gene encoding chlorophyll biosynthetic enzyme CHLL as knock-in sites, the *gfp* gene was delivered into and expressed properly in *N. oceanica* by electroporation. Moreover, the antibiotic construct harboring the *ble* gene was utilized to validate the chloroplast integration of transformants. Genotyping of the homoplasmic cells showed a site-specific recombination of the transforming cassette into chloroplast genomes.

Restrictively, herein presented proof-of principle represents a starting point of plastome engineering for *N. oceanica* where the transformation frequency remains to be improved. To ensure the frequency, a standard practice should be developed and more recombination sites and more selectable marker genes should be tested. With a streamlined practice, electroporation should facilitate plastid engineering of relevant species with relative small cell sizes or unknown cell structure of which the chlroroplast manipultion is intractable by using microparticle bombardment or PEG-mediated transformation methods. Given that FA biosynthesis and photosynthesis processes predominantly take place in chloroplasts, transplastomic technology can be utilized to created engineered microalgal strains with optimized oil production and robust photosynthetic efficiency. Moreover, the incorporation of transgenes into the plastid genome for containment and high-level expression of recombinant proteins holds great promise for pharmaceutical and industrial applications.

## Author contributions

YL: Design the research; QG, JJ, and XH: Conducted the experiments; YL and SW: Wrote the first version of the manuscript; YL: Contributed to the final writing and presentation of the data.

### Conflict of interest statement

The authors declare that the research was conducted in the absence of any commercial or financial relationships that could be construed as a potential conflict of interest.
