# Supplementary material for: Engineering the Chloroplast Genome of Oleaginous Marine Microalga Nannochloropsis oceanica
Source: Front Plant Sci. 2018 Apr 11;9:439. doi: 10.3389/fpls.2018.00439 (PMC5904192; doi:10.3389/fpls.2018.00439)
Supplement: Supplementary file 1 [file Table1.PDF]

**Table S1.** Primers used in these experiments.

| Primer | Oligonucleotide Sequence 5'–3' | Product Size (bp) |
|---|---|---|
| **chlL-F1** | GGTACCGTAAACCAAGGTCGTTCTCCA | |
| **chlL-R1** | CTCGAGCTAGAAGTAACCGCAAAT | 949 |
| **5'rbcL-F** | CTCGAGGCTTACTTATTAGCCACCACCTACA | |
| **5'rbcL-R** | AAGCTTTTAGGACTCCTTTTATATAGCAGTAAA | 566 |
| **gfp-F** | AAGCTTATGGTGAGCAAGGGCGAGGA | |
| **gfp -R** | GATATCTTACTTGTACAGCTCGTCCATGCC | 732 |
| **BLE-F** | TAAAAGCTTATGGCCAAGCTGACCAGCG | |
| **BLE-R** | GATATCTTAGTCCTGCTCCTCGGCCAC | 397 |
| **psbA-F** | GATATCGAATATTTAATTACACATGAG | |
| **psbA-R** | GGATCCAACTAGTTCATCAATTTGATGAA | 364 |
| **c2-F** | GCCACAGGTTCAAATCCTGT | |
| **c2-R** | TAATCCGCATAATTCAATGGTG | 598/1797 |
| **chlL-F2** | GGATCCATCCGAAGCATGATAGCACTT | |
| **chlL-R2** | GAGCTCCAGCTTCTTCAAGTTCAGCCA | 1050 |
