# Supplementary material for: Engineering the Chloroplast Genome of Oleaginous Marine Microalga Nannochloropsis oceanica
Source: Front Plant Sci. 2018 Apr 11;9:439. doi: 10.3389/fpls.2018.00439 (PMC5904192; doi:10.3389/fpls.2018.00439)
Supplement: Supplementary file 2 [file DataSheet1.PDF]

## Supplemental Dataset 1: sequence of pMEMc1 transforming cassette

chlL homologous recombination region (s1.31655–s1.32380; s1.32520-s1. 33456)
*rbcL* promoter (s1.26100-s1.26655)
*gfp* gene, codon optimized
*psbA* 3’UT*R* (s1.28647- s1.28954)
UNDERLINED ARE PRIMER BINDING SITES

TAACCCTCACTAAAGGGAACAAAAGCTGGTACCGTAAACCAAGGTCGTTCTCCAGTT
GGTAAACGTGATGCTTCAATTGGTGGAAACCCAAACCCAGCTTCTTTAAAGTTTCAAT
CAAATACCTAGAATTTATTTCTAGATAGTATTAAAATACATCTTAATTCATATCCTTTTACT
TTTACTTGAAGGGTATGATCTTAGGAGAGATGGCAGAGTGGTCGATTGCGTCTGACT
TGAAATCAGAAGAACTAGGAATGGTTCCGTGGGTTCGAATCCCACTCTCTCTTCTCA
ATATTTTTATTAAAAATATAGGTTGATTCTTTTGATTTAACTTTTTTCTATTAAAATATCTAA
TAGAACATTTCAAAAGTATTGTTTGGTTAACCTAAACCAGTTTATAATCTTTTAACTAAA
GAGGTATATATGTTAGTACTAAAAATAGCAGTTTACACAGTTGTTAGCTTTTTCGTTTAT
CTATTTTGGTTTGGATTTATTTCAAATGACCCGTCACGTAACCCTACACAGAATATTAAT
AACTAATTAAAAGTTTGGTATTTATCATAAGGCATATTAAATAAATAGAAATAATGCGCCT
TATGATAAACGTAAATAAGTGCTATATATAAATAAAAGGGCAGTTAGCTCAGCGGTAGA
GCTTCTGCCTTACAAGCAGAAGGCCACAGGTTCAAATCCTGTACTGCCCATAGGGCT
CATCGTCTAAGGGATTAGGACAGAAACCTTCTAAGTTTCTAATGTAGGTTCGAATCCTA
CTGGGCCTAAGACGTACTGAGTATAAAAAATTAAACATGATATTTTAAGGGTTACAGAT
AAACAAATGTTTTTGAGAAGATACTCTTTACTCCCAGAATTTAAATACTAGTTGTCTGAT
TTTTTAACTCTGACACTCTAGACCTTATATTATAGTATTTTATGAGCAATTTATAAAAAATA
AATTTGCGGTTACTTCTAGCTCGAGGCTTACTTATTAGCCACCACCTACAATTGTTACA
AATTCAATTTGATCAAAAGGATTCAAAAATATTTTTTTTGACAAATCGGGTTTTAGGATT
ACTTGGTTATATTCAGTAATTAAAACGTTATGTTTAATATCTAAAAATTCAAAGAGTTGAC
TGAGGTTAACAGTATAATTAATGGCGTAAACTTCACCATTAATTAAAACCCAATTTTCTG
GATTCGGATATAATTCATTTTTGATAAAAGTCATCGGTATTTGTACATATATAACGAATAA
TTATTATTCAGATAAATTTTAAAAATGATTAAATTTTTTATTTTAGGTCTTAAAATGACCTA
AAAATATTAAAATAGATAAAATTGTTGTATATTAAAGAAATCATTCATATAATATTTATGTAT
CTAAAATAGTTCAAATTAGAGATTTATATATTGAATATTAATTAATTTTCAATAAAATCTCA
AGTTAAATGGGTTTTGCTTTTCAAGTTCGGTTTAAATTGCCGAAGGGACCATTTAAAAT
TTCCGAGAACCAGAGAATTTACTGCTATATAAAAGGAGTCCTAAAAGCTT***ATG***GTGAG
CAAGGGCGAGGAGCTGTTCACCGGGGTGGTGCCCATCCTGGTCGAGCTGGACGGC
GACGTAAACGGCCACAAGTTCAGCGTGTCCGGCGAGGGCGAGGGCGATGCCACCT
ACGGCAAGCTGACCCTGAAGTTCATCTGCACCACCGGCAAGCTGCCCGTGCCCTG
GCCCACCCTCGTGACCACCCTGACCTACGGCGTGCAGTGCTTCAGCCGCTACCCC
GACCACATGAAGCAGCACGACTTCTTCAAGTCCGCCATGCCCGAAGGCTACGTCCA
GGAGCGCACCATCTTCTTCAAGGACGACGGCAACTACAAGACCCGCGCCGAGGTG

AAGTTCGAGGGCGACACCCTGGTGAACCGCATCGAGCTGAAGGGCATCGACTTCAA
GGAGGACGGCAACATCCTGGGGCACAAGCTGGAGTACAACTACAACAGCCACAACG
TCTATATCATGGCCGACAAGCAGAAGAACGGCATCAAGGTGAACTTCAAGATCCGCC
ACAACATCGAGGACGGCAGCGTGCAGCTCGCCGACCACTACCAGCAGAACACCCC
CATCGGCGACGGCCCCGTGCTGCTGCCCGACAACCACTACCTGAGCACCCAGTCC
GCCATGAGCAAAGACCCCAACGAGAAGCGCGATCACATGGTCCTGCTGGAGTTCGT
GACCGCCGCCGGGATCACTCTCGGCATGGACGAGCTGTACAAG***TAA***GATATCGAATA
TTTAATTACACATGAGTGATTTTTAATCACTCATGTGTAATAATTTTTTTGATCTAATTTAA
TTAAGTAAAAATGAAACGGTTAGACTTTTTTATATACTTGCTAAATTAAGTGTAAAAATAC
TATTTTATAGTATTGTTGCTCTATCCAACTTAACTGAGCGTCTAAGTATTATACTCAAACT
AACTTCATGGATAGGTAAAAAAGATAAGCGAACGATTTCAAATGGAATTTAATTTTACT
CAAAAATAAAGTGAACGCGGTATCTATACATTCTAGTAAATTATAAAAATTGATCTAATAA
AGTAAGTAAGTAACAACTAGTTCATCAAATTGATGAACTAGTTGGATCCATCCGAAGCA
TGATAGCACTTTTACATTAACAGGATTTTTAATTCCAACAATTATTGATACATTACAAGTC
AAAGATTACCACTATGAGGATGTTTGGCCTGAAGATGTAATTTATAGAGGATTTAATGG
TGTAGATTGTGTTGAAGCTGGTGGACCGCCAGCTGGAGCTGGTTGTGGTGGTTACG
TCGTTGGTGAAACTGTTAAACTTTTAAAAGAGCTTAATGCATTTGATGAATACGATGTT
ATTCTTTTTGATGTATTAGGTGATGTAGTTTGCGGTGGATTTGCAGCACCATTGAATTA
TGCGGATTACTGTTTAATTGTTACAGACAATGGCTTTGATGCCTTATTTGCCGCAAATA
GAATTGCAGCTTCGGTTCGCGAAAAAGCTAGGACTCACAGTTTAAGATTAGCTGGTT
TAATAGGTAATCGAACTGCAACACGTGATTTAATTGATAAATATATTCAAACTGTACCAA
TCCCAGTTCTTGAAGTTTTGCCACTAATTGAAGATATACGGGTTTCAAGAATTAAAGGT
AAAACGCTTTTTGAGATGTCTATAATTGATCCCTCATTGGAGTACATTTGTGATTATTAC
TTAAATATTGCAGATCAACTTATAGCTCAACCAGAAGGTGTAATTCCAAAAGAATCAGC
TGATCGTGAATTATTTACTCTTTTATCTGATTTTTATTTAAAACCCTCGGATACAGAACA
AAATTTAGACAATATGGAGATGAATCTTTTTAATAATTAAATTACGTCTTAATGAATAAAA
AAATTAAAAAGGAATTTAAATAATATGAATACAGAACAAGGTTCATTAATTAATTCTAATT
CCATTACTTTTGAATGCGAAACTGGTAATTATCATACATTTTGTCCTATAAGTTGTGTTG
CTTGGTTATATCAAAAAATTGAAGACAGTTTCTTTTTGGTAATTGGAACAAAAACGTGT
GGCTATTTCTTACAGAATGCATTAGGGGTAATGATATTTGCTGAACCAAGGTACGCAAT
GGCTGAACTTGAAGAAGCTGGAGCTCCAATTCGCCCTA

## Supplemental Dataset 2: sequence of pMEMc2 transforming cassette

chlL homologous recombination region (s1.31655–s1.32380; s1.32520-s1.33456)
*rbcL* promoter (s1.26100-s1.26655)
*ble* gene
*psbA* 3’UT*R* (s1.28647- s1.28954)
UNDERLINED ARE PRIMER BINDING SITES

TAACCCTCACTAAAGGGAACAAAAGCTGGTACCGTAAACCAAGGTCGTTCTCCAGTT
GGTAAACGTGATGCTTCAATTGGTGGAAACCCAAACCCAGCTTCTTTAAAGTTTCAAT
CAAATACCTAGAATTTATTTCTAGATAGTATTAAAATACATCTTAATTCATATCCTTTTACT
TTTACTTGAAGGGTATGATCTTAGGAGAGATGGCAGAGTGGTCGATTGCGTCTGACT
TGAAATCAGAAGAACTAGGAATGGTTCCGTGGGTTCGAATCCCACTCTCTCTTCTCA
ATATTTTTATTAAAAATATAGGTTGATTCTTTTGATTTAACTTTTTTCTATTAAAATATCTAA
TAGAACATTTCAAAAGTATTGTTTGGTTAACCTAAACCAGTTTATAATCTTTTAACTAAA
GAGGTATATATGTTAGTACTAAAAATAGCAGTTTACACAGTTGTTAGCTTTTTCGTTTAT
CTATTTTGGTTTGGATTTATTTCAAATGACCCGTCACGTAACCCTACACAGAATATTAAT
AACTAATTAAAAGTTTGGTATTTATCATAAGGCATATTAAATAAATAGAAATAATGCGCCT
TATGATAAACGTAAATAAGTGCTATATATAAATAAAAGGGCAGTTAGCTCAGCGGTAGA
GCTTCTGCCTTACAAGCAGAAGGCCACAGGTTCAAATCCTGTACTGCCCATAGGGCT
CATCGTCTAAGGGATTAGGACAGAAACCTTCTAAGTTTCTAATGTAGGTTCGAATCCTA
CTGGGCCTAAGACGTACTGAGTATAAAAAATTAAACATGATATTTTAAGGGTTACAGAT
AAACAAATGTTTTTGAGAAGATACTCTTTACTCCCAGAATTTAAATACTAGTTGTCTGAT
TTTTTAACTCTGACACTCTAGACCTTATATTATAGTATTTTATGAGCAATTTATAAAAAATA
AATTTGCGGTTACTTCTAGCTCGAGGCTTACTTATTAGCCACCACCTACAATTGTTACA
AATTCAATTTGATCAAAAGGATTCAAAAATATTTTTTTTGACAAATCGGGTTTTAGGATT
ACTTGGTTATATTCAGTAATTAAAACGTTATGTTTAATATCTAAAAATTCAAAGAGTTGAC
TGAGGTTAACAGTATAATTAATGGCGTAAACTTCACCATTAATTAAAACCCAATTTTCTG
GATTCGGATATAATTCATTTTTGATAAAAGTCATCGGTATTTGTACATATATAACGAATAA
TTATTATTCAGATAAATTTTAAAAATGATTAAATTTTTTATTTTAGGTCTTAAAATGACCTA
AAAATATTAAAATAGATAAAATTGTTGTATATTAAAGAAATCATTCATATAATATTTATGTAT
CTAAAATAGTTCAAATTAGAGATTTATATATTGAATATTAATTAATTTTCAATAAAATCTCA
AGTTAAATGGGTTTTGCTTTTCAAGTTCGGTTTAAATTGCCGAAGGGACCATTTAAAAT
TTCCGAGAACCAGAGAATTTACTGCTATATAAAAGGAGTCCTAAAAGCTT***ATG***GCCAA
GCTGACCAGCGCCGTTCCGGTGCTCACCGCGCGCGACGTCGCCGGAGCGGTCGA
GTTCTGGACCGACCGGCTCGGGTTCTCCCGGGACTTCGTGGAGGACGACTTCGCC
GGTGTGGTCCGGGACGACGTGACCCTGTTCATCAGCGCGGTCCAGGACCAGGTGG
TGCCGGACAACACCCTGGCCTGGGTGTGGGTGCGCGGCCTGGACGAGCTGTACGC
CGAGTGGTCGGAGGTCGTGTCCACGAACTTCCGGGACGCCTCCGGGCCGGCCATG
ACCGAGATCGGCGAGCAGCCGTGGGGGCGGGAGTTCGCCCTGCGCGACCCGGCC
GGCAACTGCGTGCACTTCGTGGCCGAGGAGCAGGAC***TAA***GATATCGAATATTTAATT
ACACATGAGTGATTTTTAATCACTCATGTGTAATAATTTTTTTGATCTAATTTAATTAAGT
AAAAATGAAACGGTTAGACTTTTTTATATACTTGCTAAATTAAGTGTAAAAATACTATTTT
ATAGTATTGTTGCTCTATCCAACTTAACTGAGCGTCTAAGTATTATACTCAAACTAACTT
CATGGATAGGTAAAAAAGATAAGCGAACGATTTCAAATGGAATTTAATTTTACTCAAAA
ATAAAGTGAACGCGGTATCTATACATTCTAGTAAATTATAAAAATTGATCTAATAAAGTAA
GTAAGTAACAACTAGTTCATCAAATTGATGAACTAGTTGGATCCATCCGAAGCATGATA
GCACTTTTACATTAACAGGATTTTTAATTCCAACAATTATTGATACATTACAAGTCAAAG
ATTACCACTATGAGGATGTTTGGCCTGAAGATGTAATTTATAGAGGATTTAATGGTGTA
GATTGTGTTGAAGCTGGTGGACCGCCAGCTGGAGCTGGTTGTGGTGGTTACGTCGT
TGGTGAAACTGTTAAACTTTTAAAAGAGCTTAATGCATTTGATGAATACGATGTTATTCT
TTTTGATGTATTAGGTGATGTAGTTTGCGGTGGATTTGCAGCACCATTGAATTATGCG

GATTACTGTTTAATTGTTACAGACAATGGCTTTGATGCCTTATTTGCCGCAAATAGAAT
TGCAGCTTCGGTTCGCGAAAAAGCTAGGACTCACAGTTTAAGATTAGCTGGTTTAATA
GGTAATCGAACTGCAACACGTGATTTAATTGATAAATATATTCAAACTGTACCAATCCC
AGTTCTTGAAGTTTTGCCACTAATTGAAGATATACGGGTTTCAAGAATTAAAGGTAAAA
CGCTTTTTGAGATGTCTATAATTGATCCCTCATTGGAGTACATTTGTGATTATTACTTAA
ATATTGCAGATCAACTTATAGCTCAACCAGAAGGTGTAATTCCAAAAGAATCAGCTGAT
CGTGAATTATTTACTCTTTTATCTGATTTTTATTTAAAACCCTCGGATACAGAACAAAATT
TAGACAATATGGAGATGAATCTTTTTAATAATTAAATTACGTCTTAATGAATAAAAAAATT
AAAAAGGAATTTAAATAATATGAATACAGAACAAGGTTCATTAATTAATTCTAATTCCATT
ACTTTTGAATGCGAAACTGGTAATTATCATACATTTTGTCCTATAAGTTGTGTTGCTTG
GTTATATCAAAAAATTGAAGACAGTTTCTTTTTGGTAATTGGAACAAAAACGTGTGGCT
ATTTCTTACAGAATGCATTAGGGGTAATGATATTTGCTGAACCAAGGTACGCAATGGC
TGAACTTGAAGAAGCTGGAGCTCCAATTCGCCCTA

## Supplemental Dataset 3: sequence of pMEMc2 transformants

chlL homologous recombination region (s1.31655–s1.32380; s1.32520-s1.33456)
*rbcL* promoter (s1.26100-s1.26655)
*ble* gene
*psbA* 3’UT*R* (s1.28647- s1.28954)
UNDERLINED ARE PRIMER BINDING SITES

GCCACAGGTTCAAATCCTGTACTGCCCATAGGGCTCATCGTCTAAGGGATTAGGACA
GAAACCTTCTAAGTTTCTAATGTAGGTTCGAATCCTACTGGGCCTAAGACGTACTGAG
TATAAAAAATTAAACATGATATTTTAAGGGTTACAGATAAACAAATGTTTTTGAGAAGATA
CTCTTTACTCCCAGAATTTAAATACTAGTTGTCTGATTTTTTAACTCTGACACTCTAGAC
CTTATATTATAGTATTTTATGAGCAATTTATAAAAAATAAATTTGCGGTTACTTCTAGCTC
GAGGCTTACTTATTAGCCACCACCTACAATTGTTACAAATTCAATTTGATCAAAAGGAT
TCAAAAATATTTTTTTTGACAAATCGGGTTTTAGGATTACTTGGTTATATTCAGTAATTAA
AACGTTATGTTTAATATCTAAAAATTCAAAGAGTTGACTGAGGTTAACAGTATAATTAAT
GGCGTAAACTTCACCATTAATTAAAACCCAATTTTCTGGATTCGGATATAATTCATTTTT
GATAAAAGTCATCGGTATTTGTACATATATAACGAATAATTATTATTCAGATAAATTTTAAA
AATGATTAAATTTTTTATTTTAGGTCTTAAAATGACCTAAAAATATTAAAATAGATAAAATT
GTTGTATATTAAAGAAATCATTCATATAATATTTATGTATCTAAAATAGTTCAAATTAGAGA
TTTATATATTGAATATTAATTAATTTTCAATAAAATCTCAAGTTAAATGGGTTTTGCTTTTC
AAGTTCGGTTTAAATTGCCGAAGGGACCATTTAAAATTTCCGAGAACCAGAGAATTTA
CTGCTATATAAAAGGAGTCCTAAAAGCTT***ATG***GCCAAGCTGACCAGCGCCGTTCCGG
TGCTCACCGCGCGCGACGTCGCCGGAGCGGTCGAGTTCTGGACCGACCGGCTCG
GGTTCTCCCGGGACTTCGTGGAGGACGACTTCGCCGGTGTGGTCCGGGACGACGT
GACCCTGTTCATCAGCGCGGTCCAGGACCAGGTGGTGCCGGACAACACCCTGGCC
TGGGTGTGGGTGCGCGGCCTGGACGAGCTGTACGCCGAGTGGTCGGAGGTCGTGT
CCACGAACTTCCGGGACGCCTCCGGGCCGGCCATGACCGAGATCGGCGAGCAGCC

GTGGGGGCGGGAGTTCGCCCTGCGCGACCCGGCCGGCAACTGCGTGCACTTCGT
GGCCGAGGAGCAGGAC***TAA***GATATCGAATATTTAATTACACATGAGTGATTTTTAATCA
CTCATGTGTAATAATTTTTTTGATCTAATTTAATTAAGTAAAAATGAAACGGTTAGACTTT
TTTATATACTTGCTAAATTAAGTGTAAAAATACTATTTTATAGTATTGTTGCTCTATCCAAC
TTAACTGAGCGTCTAAGTATTATACTCAAACTAACTTCATGGATAGGTAAAAAAGATAA
GCGAACGATTTCAAATGGAATTTAATTTTACTCAAAAATAAAGTGAACGCGGTATCTAT
ACATTCTAGTAAATTATAAAAATTGATCTAATAAAGTAAGTAAGTAACAACTAGTTCATCA
AATTGATGAACTAGTTGGATCCATCCGAAGCATGATAGCACTTTTACATTAACAGGATT
TTTAATTCCAACAATTATTGATACATTACAAGTCAAAGATTACCACTATGAGGATGTTTG
GCCTGAAGATGTAATTTATAGAGGATTTAATGGTGTAGATTGTGTTGAAGCTGGTGGA
CCGCCAGCTGGAGCTGGTTGTGGTGGTTACG

## Supplemental Dataset 4: sequence of wild-type *N. oceanica*

chlL region
UNDERLINED ARE PRIMER BINDING SITES
GCCACAGGTTCAAATCCTGTACTGCCCATAGGGCTCATCGTCTAAGGGATTAGGACA
GAAACCTTCTAAGTTTCTAATGTAGGTTCGAATCCTACTGGGCCTAAGACGTACTGAG
TATAAAAAATTAAACATGATATTTTAAGGGTTACAGATAAACAAATGTTTTTGAGAAGATA
CTCTTTACTCCCAGAATTTAAATACTAGTTGTCTGATTTTTTAACTCTGACACTCTAGAC
CTTATATTATAGTATTTTATGAGCAATTTATAAAAAATAAATTTGCGGTTACTTCTAGGAG
AAAAAAATGCGATTAGCAGTTTATGGTAAAGGTGGTATTGGAAAATCTACTACAAGCTG
TAATATTTCAGTAGCGTTAGCACAAAGAGGTAAAAAAGTTTTACAAATTGGATGTGATC
CGAAGCATGATAGCACTTTTACATTAACAGGATTTTTAATTCCAACAATTATTGATACAT
TACAAGTCAAAGATTACCACTATGAGGATGTTTGGCCTGAAGATGTAATTTATAGAGGA
TTTAATGGTGTAGATTGTGTTGAAGCTGGTGGACCGCCAGCTGG

## Supplemental Dataset 5: Plastid genome insertion of marker cassette

Plastid genome s1.28695- s1.29028
Promoter
marker gene *ble*

ACGGGGGTCGGTCAGACTCCACCGCTCCGGCCACGTCCCTCGCGGTCAACACAGGCACGGCGCTGGTCAACTTGGCCATC *TCG*
*A*GGGTCTTTGGTTGTGATATTGTTTTGTTACTGTGGTATGGGTGAAGGTTGGGAGCGGTGAGCGAGTCGTCGAAAGTTCTTTG
TGTTTTCTGTGGCTTTCGCTGTCCCTTTTCCATGTCGCAGAAGAACGAAAATGACACAAGGCTACAGCGGGGGCACGATGCAT
CGTCACCGTAACTGAACACGTAGTGTTATCAAGACGCATCGGCAGGGCGGGCACGCGCCATGTGGGTGAAGGCATCTTTAAAG

ACGAAGCCGCTCTCCGCTCCACAGCCACACACACAGCACGTTCTCTGCCTGAAGACGAAAATTTGACGCTTCGCAGAGGCTTG
GAGTCGTGCCAGCAGCCCCTGCTTTGTATTCTACATTGAGTGGGACAGGGTTTACGAGAATCTCTAGAGGATCCCCGGGTACC
GAGCTCGAATTCGTAATCATGGTCATAGCTGTTTCCTGTGTGAAATTGTTATCCGCTCAC*CGCCAGGGTTTTCCCAGTCACGA*
*CGTTGTAAAACGACGGCCAGTG*CCAAGCTTGCATGCCTGCAGGTCGACGATTGAATGTATAGATACCGCGTTCACTTTATTTT
TGAGTAAAATTAAATTCCATTTGAAATCGTTCGCTTATCTTTTTTACCTATCCATGAAGTTAGTTTGAGTATAATACTTAGAC
GCTCAGTTAAGTTGGATAGAGCAACAATACTATAAAATAGTATTTTTACACTTAATTTAGCAAGTATATAAAAAAGTCTAACC
GTTTCATTTTTACTTAATTAAATTAGATCAAAAAAATTATTACACATGAGTGATTAAAAATCACTCATGTGTAATTAAATATT
CTTAAGCATTAACAGCAGGAGCGTTTAATGCAACTGGAAGGACATTAGATACGGGATCCCCGGGCTGCAGGAATTCGATATCT
TAGTCCTGCTCTCGGCCACGAAGTGCACGCAGTTCCTGCAGGGTCCCTCAAGGCGAACTCCCTCCCCCAAGCTGCTCTCCGAA
TCTCGGTCATGGCAGGGCCCGAAGGCGGTTACGCAGTCTACAAGGGGCGGTCCAGACAAAAA
